# Smoking in film in New Zealand: measuring risk exposure

**DOI:** 10.1186/1471-2458-6-243

**Published:** 2006-10-04

**Authors:** Jesse Gale, Bridget Fry, Tara Smith, Ken Okawa, Anannya Chakrabarti, Damien Ah-Yen, Jesse Yi, Simon Townsend, Rebecca Carroll, Alannah Stockwell, Andrea Sievwright, Kevin Dew, George Thomson

**Affiliations:** 1 Wellington School of Medicine and Health Sciences, University of Otago, PO Box 7343, Wellington South, New Zealand

## Abstract

**Background:**

Smoking in film is a risk factor for smoking uptake in adolescence. This study aimed to quantify exposure to smoking in film received by New Zealand audiences, and evaluate potential interventions to reduce the quantity and impact of this exposure.

**Methods:**

The ten highest-grossing films in New Zealand for 2003 were each analysed independently by two viewers for smoking, smoking references and related imagery. Potential interventions were explored by reviewing relevant New Zealand legislation, and scientific literature.

**Results:**

Seven of the ten films contained at least one tobacco reference, similar to larger film samples. The majority of the 38 tobacco references involved characters smoking, most of whom were male. Smoking was associated with positive character traits, notably rebellion (which may appeal to adolescents). There appeared to be a low threshold for including smoking in film. Legislative or censorship approaches to smoking in film are currently unlikely to succeed. Anti-smoking advertising before films has promise, but experimental research is required to demonstrate cost effectiveness.

**Conclusion:**

Smoking in film warrants concern from public health advocates. In New Zealand, pre-film anti-smoking advertising appears to be the most promising immediate policy response.

## Background

The majority of adult smoking is initiated during adolescence. In the United States, 89% of 30–39 year-old regular smokers began smoking before the age of 18 [1]. In a New Zealand cohort, the prevalence of adolescent smoking increased from near nil at nine years to adult rates by age 18 [2]. The majority of New Zealand smokers start smoking before completing year 10 at school (14–15 years old) [3,4]. It appears that New Zealand children and adolescents enter into nicotine addiction without sufficient knowledge of its nature [5], and are likely to have no real knowledge at all of the speed or irreversibility of nicotine addiction [6-8]. Thus, factors that influence child and adolescent smoking uptake are important targets for smoke-free advocates.

One factor that may increase smoking uptake by New Zealand adolescents is the prominent and excessive portrayal of smoking in film and other mass media [9]. The majority of 14–17 year old New Zealanders are at least annual cinema viewers, and adolescents are over-represented amongst those who attend New Zealand cinemas more than monthly [10].

In the USA, smoking amongst major film characters was found to exceed the true societal prevalence by a factor of three [11,12]. In the 25 highest-grossing films in USA each year, from 1988 to 1997, 87% contained tobacco use, with an average of 5 occurrences per film [13]. In the top ten films of 1985–1995, 98% contained at least one "pro-tobacco event" [14]. Some analyses also suggest smoking is increasing in films since the 1980s, especially in young and female characters [11,12], but a larger sample found a small decrease throughout the 1990s [15]. Exposure to smoking in film received by New Zealand audiences has not yet been published.

A brief review of international research indicates that exposure to smoking in film influences smoking uptake by adolescents. For instance, a cohort of 3547 American 10–14 year-olds, followed for 13–26 months were more than twice as likely to begin smoking if exposed to smoking in film.[16] Over half of the 10% smoking uptake by this cohort during follow up was attributed to exposure to smoking in film. Those whose parents allowed them to watch R-rated films more than 'once in a while' were 2.8 times more likely to try smoking during follow up.[17] A cross-sectional study by the same group found exposure to smoking in film was associated with doubled odds of trying smoking, before and after adjustment for 15 covariant predictors of smoking initiation.[18] In other cross-sectional studies, the use of tobacco by popular actors on screen had the greatest impact on smoking initiation behaviour in adolescents.[19,20] Although prospective data are few, it would seem that smoking in film may be an important and preventable risk for smoking behaviour.

Adolescent perceptions of smoking on-screen are important to the translation of smoking viewing to smoking behaviour. Focus group research with 165 New Zealand adolescents indicates that on-screen smoking reinforced inaccurate perceptions of smoking as ubiquitous, normal, and acceptable.[21,22] A cross-sectional survey of over 3000 New Zealand students (age 12–17) indicated that image based associations (e.g. smoking representing sexiness, stylishness) held a greater influence on smoking behaviour than emotional associations (e.g. smoking representing relaxation, anxiety).[23] Thus the emotional context and portrayal of smoking in film is also an important factor in smoking prevention.

This study aimed to measure the exposure to smoking in film that young New Zealand audiences receive, and to briefly review possible interventions (legislative and counter campaigns) that might be used to reduce the exposure to, or impact of, smoking in film. These New Zealand data were intended to guide recommendations for smokefree advocates.

## Methods

### Film sample

The ten highest-grossing films in New Zealand for 2003 were identified with help from the Val Morgan Cinema Network, and are listed in Table 1 in order of their box office takings. These movies accounted for 38% of the New Zealand gross box office takings in 2003.

### Analysis tool

The content analysis tool used in a previous New Zealand study of smoking on television was adapted for use with films.[24] A smoking reference was defined as any on-screen smoking, smoking related imagery (e.g. ashtrays, tobacco advertising) or smoking related conversation (or combination). Every reference was timed, and details of characters (gender, age, ethnicity, social class), setting, and events, recorded. Characters' demographic data were estimated by viewers, and fantasy characters (e.g. hobbits) were assigned demographic characteristics of societal groups that they best represented (e.g. middle class, white, 15–20 year olds). Subjective qualitative data were also collected on each smoking reference: roles of smoking characters; imagery and associations; the emotional portrayal of smoking in terms of character traits, plot details and themes; and the subjective necessity of smoking for each scene. These data were interpretative and qualitative, and viewers made a succinct yet full description, but did not apply categories or pre-definitions.

### Film viewing

Five viewers were trained to use the content analysis tool consistently. Each film was viewed independently by two viewers. Where there was disagreement, the scene was reappraised and a consensus gained. In the same way, the qualitative data were discussed and agreed.

### Intervention review

All relevant New Zealand legislation was reviewed with the view to exploring opportunities to reduce smoking in film. The literature on anti-smoking interventions in film was reviewed, following a search of the Medline database. The search used keywords: film, smoking, policy, laws, and advertisements. The interventions were examined using the criteria of practicality (political, legal and cost) and effectiveness in reducing smoking uptake or increasing quitting.

## Results

### Smoking in film in New Zealand

Overall, seven (70%) of the sample films contained at least one smoking reference. There were a total of 38 smoking references: an average of 3.8 references per film. These references lasted for an average of 43.4 s per film (11.4 s per reference).

Table 1 outlines the number and types of smoking references in each film and in total, and the overall duration of these episodes.

### Smoking associations

Of the 38 smoking references, four were imagery unrelated to any specific character. The 22 different characters associated with smoking in the remaining episodes are described in Table 2.

Cigarettes were the most commonly smoked tobacco product (12/22, 54.5% of episodes), followed by pipes (6/22, 27.3%) and cigars (4/22, 18.2%). Half of the on-screen smoking was shown indoors, and 17/22 episodes showed second hand smoke (SHS) exposure. The 14 episodes of smoking-related imagery besides smoking, comprised cigarette lighters (64.3%), ashtrays (21.4%), and solitary instances of cigarette packets and a tobacco pouch (each 7.1%).

Amongst the seven films with smoking references, numerous positive associations were made: suaveness, friendship and social inclusion, and thoughtfulness. There were occasional negative themes, but these were not prominent, and none of the films portrayed smoking negatively overall. The occasional explicit anti-smoking messages were often undermined by implied messages that smoking is normal, inevitable or rebellious. Smoking was commonly associated with the character traits of rebellion and independence, which may be seen most positively by adolescent viewers (whose development involves establishing independence).

The smoking in these films appeared to be used by filmmakers for the portrayal of character traits, as opposed to plot or theme development. However, these character traits did not require smoking for expression. Sometimes the use of smoking appeared to serve no clear purpose, and there appeared to be a low threshold for including smoking in these films.

### New Zealand legislation affecting smoking in film

The Smoke-free Environment Act (1990) banned remaining tobacco advertising and sponsorship from all domains, other than products at point of sale and internationally broadcasted or distributed material. However, film content is not subject to New Zealand legislation besides the Films, Videos, and Publications Classification Act (1993). The process of film classification and labelling as established by this act has a number of limitations that make classification of films based on smoking an unlikely prospect:

1. The official classification of films depends very largely on Australian or British labelling authorities. Neither foreign authority considers smoking in their labelling. Only films that are restricted, objectionable, or difficult to classify are currently sent for consideration by the New Zealand Classification Office.

2. The criteria for film restriction in the Act are "matters of sex, horror, crime, cruelty or violence" to an extent considered "injurious to the public good." Restricted films are "likely to cause harm ... if people outside the restricted audience view [them]."

3. The New Zealand Bill of Rights Act (1990) protects freedom of expression, and works alongside the Classification Act. Where doubt remains over the likelihood of injury to the public good, freedom of expression prevails. Public health advocates would argue that smoking in film causes public harm, but this would be contested by film makers.

4. Underage smoking is criminal, and thus is considered under the Classification Act. However, only the promotion or encouraging of criminal acts are grounds for restriction.

5. Previous attempts to introduce classification based on smoking have failed. Potential legislation requires public support, and New Zealanders tend not to be supportive of specific interventions that restrict freedom.[25]

Thus legal approaches to minimise exposure to smoking in film in New Zealand would demand legislative change, considerable public education, and large increases in the number of film classifications performed in New Zealand.

### Measures to reduce harm from smoking in film

The options for interventions *within cinemas *for films with smoking, to discourage smoking uptake and improve quitting, include: health ratings or warnings for the films, mandated by legislation; or paid or required advertising about smoking risks before the films. We found no other substantive interventions suggested by the literature.

Health ratings or warnings, or required informational messages, would require new legislation. This would probably require strong evidence of the link between exposure to smoking in films and smoking initiation. There is currently no evidence for the efficacy of such warnings. Indeed, it has been shown that warning labels increase interest in violent television programs by presenting the program as a 'forbidden fruit'.[26] More research is required to assess the impact of such messages.

Research shows that anti-smoking advertising before films is a potentially effective intervention. Pechmann and Shih (1999) showed that a 30 second anti-smoking advertisement prior to a single film affected students' perceptions of smokers, smokers' self-perceptions, and reduced non-smokers' intention to smoke.[27] However, this study was performed in an American classroom setting. Edwards and colleagues (2004) studied 2036 female adolescents in Australia in real cinemas.[28] Pre-film anti-smoking advertisements significantly increased non-smokers' disapproval of smoking in the film, but did not change non-smokers' intentions to smoke (95% not intending to smoke in both exposure groups). The advertisement significantly increased intentions to quit amongst smoking adolescents.

The choice of advertising message is important in determining the effect of the intervention. Only three of the seven common message themes in anti-smoking advertising reduced American adolescents' intentions to smoke.[29] These messages demonstrated that smoking was socially unacceptable. Advertisements that stressed how bad smoking was for your health had no effect on adolescents' smoking intentions. Indeed, those adolescents who felt immune to the health risks had *stronger *intentions to smoke after viewing the advertisement, perhaps because smoking was portrayed as risk-taking and rebellious.[29] However, in contrast, the *Every cigarette is doing you damage *campaign, which evoked strong negative emotions, was shown to decrease intention to smoke and increasing quitting intentions amongst 3000 Australian adolescents.[30]

## Discussion

### The level of smoking in films seen in New Zealand

The use of an adapted content analysis tool on this small sample of films seen in New Zealand yielded a number of conclusions.

The proportion of films containing smoking references in our sample (70%) was similar to other samples (89%[31]; 87%[13]; 75%[15]). The average number of smoking references (3.8 per film), and average duration of these references (43 s) were somewhat lower than recent reported samples (5 episodes per film, 84 s duration; [13] 22 incidents per film[15]) Unlike these studies, there were no restricted films (which have higher smoking content) in our small sample. Thompson and Yokota (2001) found a similar duration of smoking (42 s per film) in their review of all G-rated animated films from 1937 to 2000.[32]

The smoking portrayed in our sample was of a similar nature to previous studied samples. The majority of the smoking references were onscreen smoking, which has been found to promote smoking behaviour most.[19] The most common smoking implement was a cigarette (57.7%), followed by pipes and then cigars. Our small sample contained two of the Lord of the Rings Trilogy, which inflated the use of pipes compared to previous film samples.[13] Cigarettes are the most accessible tobacco product for adolescents and are potentially most likely to normalise tobacco use. The characters who smoked in our sample were predominantly male (approximately 80%), but generalisations about age, class or ethnicity were difficult to make without a control (non-smoking) character sample. Notable however, was that over 30% of smoking characters appeared to be under 20 years old. Dalton et al (2002) found no association between tobacco use and the age, race, or socio-economic status.[13] Hazan and colleagues (1994) found trends toward increasing black, female, and young smoking characters, but these were still the minority.[11]

The similarity of our sample to larger international samples implies that previous research on smoking in films may be valid in New Zealand.

A large sample of 1990s films has recently contradicted previous reports of increasing smoking in film.[15] However, this larger sample contains more restricted films, and fewer mainstream films, which reduce the validity to adolescent audiences. Smoking in films remains at a level shown to promote smoking uptake.

### The effect of exposure to smoking in films

The effect of smoking in film is to normalise the behaviour amongst adolescents and create positive image associations, both of which promote smoking uptake.[21,22] Any smoking, even when portrayed in strong anti-smoking and negative emotional themes, could support the normalisation of tobacco use for young viewers. Smoke-free films might therefore be 'healthier' than anti-smoking films. This was illustrated in at least three references from our sample, where smokers are reprimanded, but the effect of the scene was to undermine smoking cessation messages, and reinforce themes such as rebellion or inevitability. Indeed, the films frequently used smoking to support positive character associations (notably thoughtfulness, friendship, and suaveness).

### Reasons for including smoking in films

Shields et al. (1999) interviewed Hollywood entertainment industry members, and the most cited reason for the inclusion of tobacco was to reveal character aspects, which is consistent with our investigation.[33] Realism was cited as a major reason for including smoking, but in the present sample numerous bar scenes were smoke free, and only one had ashtrays (so smoke-free bars can be sufficiently realistic). On the other hand, the two most realistic films (Whale Rider and Charlie's Angels: Full Throttle) both featured smoking.

While none of the films had negative overall portrayals of smoking, it is positive from a health perspective that the three smoke-free films had theoretical opportunities for smoking, but elected not to use them.

### Second-hand smoke in films

Most of the smoking episodes (77.3%) also portrayed second-hand smoking (SHS). Second hand smoke is a modifiable smoking behaviour that causes mortality and morbidity in non-smokers.[34] Thus, these films reinforce and normalise one of the least-acceptable of smoking behaviours. In at least two film scenes second hand smoke recipients were children. Although on occasion there are complaints about SHS in the films (X-Men 2, Whale Rider) generally the smoke is ignored.

### The options to reduce the harm from exposure to smoking in films

Legislative changes to reduce or restrict smoking in films shown in New Zealand currently appear impractical and unlikely to succeed, due to public and film industry opposition. Systematic investigation of public perceptions on media, health and censorship would be useful in determining the best response to these barriers. A public education campaign might improve the environment for legislative and industry-based changes.

Compared to legislation, appropriately placed information to cinema viewers appears to be a more promising option in the immediate future to reduce the impact of smoking in film. An example is pre-film anti-smoking advertising in cinemas. Careful design of advertisements is critical to the success of these campaigns.[29] Such campaigns have been shown to increase disapproval of smoking in non-smoking adolescents, and increase smokers' intentions to quit (but not decrease intention to start).[28]

In the absence of evidence that such campaigns prevent smoking uptake (which is their intent), a controlled trial is perhaps the most appropriate next step. Possible units for comparison are two otherwise similar but geographically distant cities, as the targets of this intervention are societal perceptions. There is relatively little contact of adolescents between distant cities, but most media influences are more homogenous. Six years of intervention and follow up would be appropriate to assess a cohort from 12 to 18 years of age (when smoking uptake is largely determined). The costs of such an intervention (no more than $100,000 per year in Wellington, for example) are modest compared to tobacco tax revenue or the societal costs from smoking uptake. There would be a reasonable expectation of public health benefit.

## Conclusion

The best strategies to address this important public health concern are currently unclear. Public support is critical to most interventions, and an analysis of public perception is warranted. This might highlight the need for wider education into the health effects of mass media.[35].

## Competing interests

The author(s) declare they have no competing interests.

## Authors' contributions

JG, BF, RC, TS, KO viewed films, and performed statistical analyses. DA-Y, JY, ST, AC, ASt and ASi reviewed literature and possible interventions. KD and GT conceived of the study and its design. All authors contributed to drafting, and then reviewed and approved the final manuscript.

## Pre-publication history

The pre-publication history for this paper can be accessed here:



## Figures and Tables

**Table 1:** Smoking references in the ten highest-grossing films in New Zealand in 2003

Film	Rating Label*	Any on-screen smoking	Smoking-related imagery	Smoking related dialogue	Imagery and dialogue	Total number of references	Total duration (s)
Lord of the Rings: The Two Towers	M	2				2	10
Harry Potter: Chamber of Secrets	PG					0	0
Lord of the Rings: The Return of the King	M	4			1	5	82
Matrix Reloaded	M	2				2	5
Whale Rider	PG	5		2		7	92
Finding Nemo	PG					0	0
Pirates of the Caribbean	M					0	0
Terminator 3: Rise of the Machines	M		1			1	10
X-Men 2	M	4	6		2	12	197
Charlies Angels: Full Throttle	M	5	4			9	38
**Total**		**22**	**11**	**2**	**3**	**38**	**434**

* Neither PG nor M ratings prevent young children from viewing, but PG recommends parental guidance for younger viewers, and M are recommended for mature audiences (16 years and over).

**Table 2:** Demographics of characters associated with smoking references

	Number (percentage) of characters (n = 22)	
Male	18	(81.8%)
Female	4	(18.2%)
**Age Group (years)**		
10–15	1	(4.5%)
16–20	6	(27.3%)
21–30	5	(22.7%)
31–40	2	(9.1%)
41–50	6	(27.3%)
≥ 51	2	(9.1%)
**Ethnic Group**		
European	15	(68.2%)
Māori	6	(27.3%)
Mongolian	1	(4.5%)
**Social Class**		
Lower	7	(31.8%)
Middle	9	(40.9%)
Upper	6	(27.3%)
